# *In Vitro* Studies on the Genotoxic Effects of Wood Smoke Flavors

**DOI:** 10.5487/TR.2008.24.4.321

**Published:** 2008-12-01

**Authors:** Young-Shin Chung, Jun-Ho Ahn, Ki-Hwan Eum, Seon-A Choi, Se-Wook Oh, Yun-Ji Kim, Sue Nie Park, Young-Na Yum, Joo-Hwan Kim, Michael Lee

**Affiliations:** 111 Medvill Co., Ltd., Seoul, 153-801 Korea; 211 Department of Biology, College of Natural Sciences, University of Incheon, 177 Dowhadong, Nam-gu, Incheon, 402-749 Korea; 311 Korea Food Research Institute, Sungnam, 463-746 Korea; 411 Division of Genetic Toxicology, National Institute of Toxicological Research, Korea Food and Drug Administration, Seoul, 122-704 Korea

**Keywords:** Wood smoke flavors, Ames assay, Chromosomal aberration test, Micronucleus assay, Comet assay, Genotoxicity

## Abstract

Smoke flavors based on the thermal decomposition of wood have been applied to a variety of food products as an alternative for traditional smoking. Despite its increasing use, the available genotoxicity data on wood smoke flavors (WSF) are still controversial. Thus, potential genotoxic effects of WSF in four short-term *in vitro* genotoxicity assays were investigated, which included the Ames assay, chromosomal aberration assay, micronucleus test and the alkaline comet assay. WSF did not cause any mutation in the Ames assay using five tester strains at six concentrations of 0.16, 0.31, 0.63, 1.25, 2.5 and 5 µl/plate. To assess clastogenic effect, the *in vitro* chromosomal aberration assay was performed using Chinese hamster lung cells. No statistically significant increase in the number of metaphases with structural aberrations was observed at the concentrations of 1.25, 2.5, and 5 µl/ml. The *in vitro* comet assay and micronucleus test results obtained on L5178Y cells also revealed that WSF has no genotoxicity potential, although there was a marginal increase in micronuclei frequencies and DNA damage in the respective micronucleus and comet assays. Taken together, based on the results obtained from these four *in vitro* studies, it is concluded that WSF is not a mutagenic agent in bacterial cells and causes no chromosomal and DNA damage in mammalian cells *in vitro*.

## INTRODUCTION

Since an invention of liquid smoke flavor prepared from primary smoke condensate (Pszczola, 1995), smoke flavorings have been applied to a variety of food products for more than 30 years. Smoke flavors are normally based on the thermal decomposition of wood, although attempts have also been made to synthesis smoke flavors by mixing chemically pure substances (Pszczola, 1995). However, smoke from burning wood contains many kinds of toxic compounds including phenols, alcohols and polycyclic aromatic hydrocarbons (PAHs), indicating that the composition of smoke depends on the material and combustion conditions (Simon *et al.*, 2005). Especially, PAH has been demonstrated to be carcinogenic and mutagenic (Brooks, 1977). Thus, EU regulation 2065/2003 on the production of smoke flavorings to be used on food was issued by the European Commission to ensure safe products (European Commission, 2003).

Despite its increasing use, the available genotoxicity data on WSF are still controversial. Several studies have been published on the mutagenicity and carcinogenicity of wood smoke flavors. Braun *et al.* (1987) reported wood smoke extracts were mutagenic in cultured human lymphoblasts but not mutagenic in a bacterial assay. Conversely, other laboratories indicated that the smoke condensates were mutagenic in a dose-related manner in mutation assays with *S. typhimurium* (Asita *et al.*, 1991; Putnam *et al.*, 1999). Thus, it is of interest to determine whether wood smoke flavors used as a food additives in Korea contains detectable levels of cell mutagens. However, no single assay is capable of detecting all relevant genotoxic agents. Therefore, the genotoxic effects of WSF were evaluated with four *in vitro* genotoxic endpoints: (1) Bacterial reverse mutation test (Ames test), (2) *In vitro* chromosome aberration test using CHL cells, (3) *In vitro* micronucleus test using L5178Y cells and (4) *In vitro* comet assay using L5178Y cells.

## MATERIALS AND METHODS

***Test item material and chemicals.*** Wood smoke flavors (WSF) in circulation in Korea was purchased in liquid condensates at a local marketplace. Chemical and microorganism inspection on element standard of WSF was performed by Korea Advanced Food Research Institute (Seoul, Korea) according to Korean Food Additives Code. The liquid smoke flavors were serially diluted to the appropriate concentrations in distilled water immediately before use. Most chemicals including positive controls such as 4-nitroquinoline 1-oxide (NQO) and cyclophosphamide were obtained from Sigma (St. Louis, MO). MEM medium, RPMI1640 medium, fetal bovine serum, and penicillin-streptomycin were purchased from GIBCO-Invitrogen (Carlsbad, CA). S9, which was prepared from male Sprague-Dawley rats induced with Aroclor 1254, was from Molecular Toxicology Inc. (Boone, NC) and cofactor for S9 mix was from Wako Pure Chem. Ind., Ltd. (Japan).

***Bacterial reverse mutation assay.*** *Salmonella typhimurium* strains TA 98 and TA1537 (detect frame-shift mutagens), and strains TA100, TA1535 and *Escherichia coli* WP2 *uvrA* (detect base-pair substitution mutagens) were used as tester strains. All of the tester strains were purchased from Molecular Toxicology Inc. (Boone, NC). The mutation assay was performed according to the method of Chung *et al.* (2004) and Maron and Ames (1983). A 0.1 ml aliquot of WSF containing 0.16~5 μl per plate, 0.5 ml of S9 mix (or sodium-phosphate buffer, pH 7.4 for S9 negative group), and 0.1 ml inoculum of the tester strain were added to each tube containing 2 ml of top agar. The contents of test tubes were mixed well and the mixtures were poured onto the Vogel-Bonner minimal agar plates. Plates were incubated at 37°C for 48 h. Triplicate plates were run for each assay.

***In vitro chromosomal aberration assay.*** *In vitro* chromosomal aberration assay was performed using Chinese hamster lung fibroblast cells (CHL) (ATCC #CRL-1935), which were obtained from American Type Culture Collection (ATCC, Manassas, VA), as described by Hong *et al.* (2005) and Dean and Danford (1984) with minor modifications. The CHL cells have a stable kayrotype, a short generation time and are easy to maintain. The assay was consisted of short-term (6 h) and continuous (24 h) treatments. Approximately 22 hours after the start of the treatment, colcemid was added to each culture at a final concentration of 0.25 μg/ml. The slides of CHL cells were prepared following the hypotonic-methanol-glacial acetic acid-flame drying-Giemsa schedule for metaphase plate analysis. The 200 metaphases (100 metaphases from each duplicate culture) were selected and analyzed for each treatment group under 1000 × magnification using a light microscope. The results were expressed as mean aberrant metaphases excluding gaps per 100 metaphases. A valid test required the aberration frequencies of the solvent controls to be within the historical range of the laboratory.

***In vitro micronucleus test.*** The micronucleus assay was performed according to Kirsch-Volders *et al.* (2003) with modifications (Oliver *et al.*, 2006). The L5178Y $tk^{+/-}$ cell line (ATCC #CRL-9518, subclone 3.7.2-C) used in this study was provided by ATCC. The day before treatment, L5178Y mouse lymphoma cells were seeded at $2 \times 10^5$ cells/ml. Cells were treated with the compound for 3 h and harvested after a 21-h recovery period, or treated for 24-h and harvested immediately. The cellular suspension was centrifuged at 1000 rpm for 5 min and cells were then resuspended in a KCl 0.075 M solution maintained at room temperature for 10 min (mild hypotonic treatment). The fixation step with methanol/acetic acid (3 : 1) solution was repeated twice and finally, cells were resuspended in a small volume of methanol/acetic acid and dropped on to clean slides. The slides were stained with 10% Giemsa (pH 6.8). Relative growth was used to assess cytotoxicity, and no cells with reduced cell growth > 30% were scored. Micronuclei were counted in 2000 cells with well-preserved cytoplasm. For a valid test, the negative control had to have < 5%. Mitomycin C and colchicine were used as direct-acting positive controls, and cyclophosphamide was used in the presence of S9 as an indirect-acting positive control. The identification of micronuclei was carried out according to Fenech (2000).

***In vitro comet assay.*** Exponentially growing L5178Y $tk^{+/}$ 3.7.2-C cells were seeded at $2 \times 10^5$ cells in 12-well plates and cultured for 24 h prior to WSF treatment, which was carried out for 3 h or 24 h with the indicated concentrations of 1.25, 2.5 and 5 μl/ml. Following drug treatment, cells were rinsed twice and resuspended at $2 \times 10^5$ cells/ml in ice-cold PBS. The comet assay was performed as described by Kim *et al.* (2006) and manufacturer's instruction. Briefly, cell suspension (25 μl) was mixed 1 : 10 with 250 μl molten low melting point (LMP) agarose, and samples of 75 μl of the mixture were rapidly spread on CometSlide™ (Trevigen, Gaithersburg, MD). After gelling for 20 min at 4°C in the dark, slides were put in a tank filled with lysis solution (2.5 M NaCl, 0.1 M EDTA, 10 mM Tris base, 1% sodium lauryl sarcosinate and 1% Triton X-100) for 1 h at 4°C in the dark. Slides were then washed three times with neutralization buffer (0.4 M Tris, pH 7.5) for 5 min and incubated in fresh alkaline buffer (0.3 M NaOH and 1 mM EDTA, pH > 13) for 30 min at room temperature to allow unwinding of DNA. Electrophoresis was then carried out at room temperature in fresh ice-cold alkaline electrophoresis buffer for 30 min (1 V/cm; 300 mA). After electrophoresis, slides were gently washed three times for 5 min in fresh neutralization buffer and exposed to 70% ethanol for 5 min. After drying at room temperature, slides were stained with 25 μl of ethidium bromide solution (20 μg/ml). Comets were examined at 200 × magnification using a fluorescence microscope (excitation filter, 515~560 nm; barrier filter, 590 nm) connected to a CCD camera. Images of 25 randomly selected nuclei per slide (two slides/culture, duplicate/dose) were analyzed using image-analysis software (Komet 5.0, Kinetic Imaging, Liverpool, UK). Tail Intensity (% of tail DNA) was used as the measure of DNA damage. The results are expressed as the mean ± SD (standard deviation). Methyl methanesulfonate was the direct-acting positive control, and cyclophosphamide was the indirect-acting positive control.

***Statistical analysis.*** The statistical analyses for *in vitro* chromosomal aberration and *in vitro* micronucleus results were conducted using Statistical Analysis System (SAS) program according to Richardson *et al.* (1989). A significant increase in micronuclei at any one concentration was determined based on a $p < 0.05$ from a one-tailed Fisher's exact test pair-wise comparison of each treatment group to control. A concentration-related response was determined based on a $p < 0.05$ from a one-tailed trend test. In CA test, pair-wise analyses of the percent aberrant cells in treated and control cultures were performed using Fisher's exact test. The result was judged as positive when there was a statistically significant and dose-related increase or a reproducible increase in the frequency of micronucleated cells (*in vitro* MN assay) or aberrant metaphases (*in vitro* CA assay). Statistically significant values that did not exceed the range of historic solvent control values were not considered positive. For statistical analysis of comet assay, the homogeneity of variances of data was tested with *Bartlett's test* ($p < 0.05$). If the variances of data were not equal, nonparametric *Kruskal-Wallis test* was used for statistical evaluations ($p < 0.05$ and 0.01). In case of Ames test, the previous results (McCann *et al.*, 1984) reported that statistical tests tend to identify more experiments as positive than considering the linear dose-response and a 2-fold increase over the spontaneous background for significance. The OECD Test Guideline 471 (OECD, 1997) also suggested that biological relevance of the results should be considered first and statistical methods might be used as an aid in evaluating the test results. Thus, no statistical analysis was performed on Ames results.

## RESULTS

***Ames assay of Wood smoke flavors (WSF).*** Histidine-requiring mutants of *S. typhimurium* (TA98, TA100, TA1535 and TA1537) and tryptophan-requiring mutants of *E. coli* WP2 *uvrA* with and without metabolic activation (S9) were used for point-mutation tests. The dose range-finding test was performed to determine the highest concentration for Ames test. No toxicity was observed after the addition of liquid condensates of WSF up to 5 μl/plate in any strains. Thus, the mutagenicity of WSF was evaluated up to a maximum dose of 5 μl/plate. The results of Ames test of WSF were shown in Table 1. There was no increase in the number of revertant colonies compared to its negative control at any dose in all of strains tested. The number of revertant colonies in both the negative and positive controls was within the range based on our historical data (data not shown).

***In vitro chromosomal aberration assay of Wood smoke flavors.*** The dose range-finding tests with the highest concentration of 5 μl/ml was performed to determine concentration of 50% growth inhibition of the cell. Duplicate treatments were conducted for each concentration. WSF had no effect on cell growth up to a maximum dose of 5 μl/ml, which was selected as the highest concentration of the main study. No statistically significant increase in the number of metaphases with structural aberrations was observed at any concentration tested both in the presence and absence of S-9 mix (Table 2). As expected, there was a significant increase in the number of aberrant metaphase both in the positive controls, cyclophosphamide (CPA) treatment for 6h+S and mitomycin C (MMC) treatment for

**Table 1.** Ames assay for wood smoke flavors (WSF)

| Tester strain | Chemical treated | Dose (µl/plate) | Without S-9 mix | | With S-9 mix | |
|---|---|---|---|---|---|---|
| | | | Colonies/plate (Mean) | [Factor][a] | Colonies/plate (Mean) | [Factor] |
| TA100 | WSF | 0 | 146 ± 17 | [1.0] | 154 ± 24 | [1.0] |
| | | 0.16 | 141 ± 7 | [1.0] | 140 ± 10 | [0.9] |
| | | 0.31 | 133 ± 4 | [0.9] | 142 ± 8 | [0.9] |
| | | 0.63 | 137 ± 14 | [0.9] | 158 ± 7 | [1.0] |
| | | 1.25 | 136 ± 7 | [0.9] | 150 ± 11 | [1.0] |
| | | 2.5 | 144 ± 19 | [1.0] | 137 ± 19 | [0.9] |
| | | 5 | 154 ± 7 | [1.1] | 162 ± 4 | [1.1] |
| TA1535 | WSF | 0 | 21 ± 3 | [1.0] | 17 ± 3 | [1.0] |
| | | 0.16 | 16 ± 5 | [0.8] | 21 ± 3 | [1.2] |
| | | 0.31 | 18 ± 4 | [0.9] | 16 ± 2 | [0.9] |
| | | 0.63 | 17 ± 2 | [0.8] | 21 ± 5 | [1.2] |
| | | 1.25 | 17 ± 8 | [0.8] | 19 ± 1 | [1.1] |
| | | 2.5 | 15 ± 5 | [0.7] | 18 ± 5 | [1.1] |
| | | 5 | 15 ± 6 | [0.7] | 17 ± 6 | [1.0] |
| TA98 | WSF | 0 | 17 ± 1 | [1.0] | 37 ± 5 | [1.0] |
| | | 0.16 | 16 ± 5 | [0.9] | 42 ± 7 | [1.1] |
| | | 0.31 | 14 ± 4 | [0.8] | 31 ± 5 | [0.8] |
| | | 0.63 | 13 ± 4 | [0.8] | 33 ± 5 | [0.9] |
| | | 1.25 | 18 ± 7 | [1.1] | 41 ± 6 | [1.1] |
| | | 2.5 | 16 ± 4 | [0.9] | 29 ± 4 | [0.8] |
| | | 5 | 13 ± 2 | [0.8] | 32 ± 5 | [0.9] |
| TA1537 | WSF | 0 | 8 ± 3 | [1.0] | 9 ± 3 | [1.0] |
| | | 0.16 | 13 ± 3 | [1.6] | 12 ± 3 | [1.3] |
| | | 0.31 | 8 ± 3 | [1.0] | 11 ± 7 | [1.2] |
| | | 0.63 | 6 ± 4 | [0.8] | 16 ± 5 | [1.8] |
| | | 1.25 | 7 ± 2 | [0.9] | 10 ± 4 | [1.1] |
| | | 2.5 | 7 ± 2 | [0.9] | 12 ± 4 | [1.3] |
| | | 5 | 6 ± 2 | [0.8] | 11 ± 1 | [1.2] |
| *E. coli* WP2 *uvrA* | WSF | 0 | 27 ± 6 | [1.0] | 28 ± 7 | [1.0] |
| | | 0.16 | 32 ± 3 | [1.2] | 33 ± 6 | [1.2] |
| | | 0.31 | 34 ± 6 | [1.3] | 40 ± 9 | [1.4] |
| | | 0.63 | 31 ± 10 | [1.1] | 34 ± 6 | [1.2] |
| | | 1.25 | 30 ± 7 | [1.1] | 34 ± 4 | [1.2] |
| | | 2.5 | 39 ± 3 | [1.4] | 40 ± 6 | [1.4] |
| | | 5 | 33 ± 3 | [1.2] | 31 ± 6 | [1.1] |
| TA100 | SA | 1.0 | 483 ± 93 | [3.3] | | |
| TA1535 | SA | 1.0 | 498 ± 56 | [23.7] | | |
| TA98 | 2-NF | 1.0 | 187 ± 4 | [11.0] | | |
| TA1537 | 9-AA | 80 | 516 ± 52 | [64.5] | | |
| WP2 *uvrA* | AF-2 | 0.01 | 191 ± 5 | [7.1] | | |
| TA100 | 2-AA | 1.0 | | | 448 ± 63 | [2.9] |
| TA1535 | 2-AA | 2.0 | | | 132 ± 9 | [7.8] |
| TA98 | 2-AA | 0.5 | | | 259 ± 23 | [7.0] |
| TA1537 | 2-AA | 2.0 | | | 797 ± 17 | [88.6] |
| WP2 *uvrA* | 2-AA | 20.0 | | | 470 ± 71 | [16.8] |

[a]No. of colonies of treated plate/No. of colonies of negative control plate.
SA, Sodium azide; 9-AA, 9-Aminoacridine; 2-AA, 2-Aminoanthracene; 2-NF, 2-Nitrofluorene; AF-2, 2-Aminofluorene.

6h-S and 24h-S. The number of metaphases with structural aberrations in the vehicle and positive control groups were within the range established in historical data. Thus, WSF was considered not to be clastogenic in this assay at up to the highest feasible concentration which could be evaluated.

***In vitro* micronucleus assay of wood smoke flavors.** Table 3 shows the results related to the evaluation of WSF in *in vitro* micronucleus test. The posi-

**Table 2.** *In vitro* chromosomal aberration assay for wood smoke flavors

| Conc. (μl/ml) | S9 mix | Times[a] (hours) | Aberrant metaphases excluding gaps (%) | Aberrant metaphases including gaps (%) |
|---|---|---|---|---|
| 6 h treatment | | | | |
| 0 | + | 6-18 | 0.0[b] | 1.5 |
| 1.25 | + | 6-18 | 0.0 | 3.0 |
| 2.5 | + | 6-18 | 0.0 | 0.5 |
| 5 | + | 6-18 | 0.5 | 1.5 |
| CPA 5 | + | 6-18 | 37.0**[c] | 44.0 |
| 6 h treatment | | | | |
| 0 | - | 6-18 | 0.0 | 2.0 |
| 1.25 | - | 6-18 | 0.0 | 1.0 |
| 2.5 | - | 6-18 | 0.0 | 2.5 |
| 5 | - | 6-18 | 0.0 | 2.5 |
| MMC 0.1 | - | 6-18 | 10.0** | 11.0 |
| 24 h treatment | | | | |
| 0 | - | 24-0 | 0.0 | 1.0 |
| 1.25 | - | 24-0 | 0.0 | 1.5 |
| 2.5 | - | 24-0 | 1.0 | 2.5 |
| 5 | - | 24-0 | 0.0 | 0.5 |
| MMC 0.1 | - | 24-0 | 26.5** | 30.5 |

[a]Time, Chemical treatment time-recovery time.
[b]Means of duplicate cultures; 100 metaphases were examined per culture.
[c]Fisher's exact test; ** significantly different from the control at $p < 0.01$.
Abbreviation: CPA, cyclophosphamide monohydrate; MMC, mitomycin C.

**Table 3.** *In vitro* micronucleus assay for wood smoke flavors

| Conc. (μl/ml) | S9 mix | Times[a] (hours) | Mean Micronuclei/1000 cells[b] | Relative cell count (%)[c] |
|---|---|---|---|---|
| 3 h treatment | | | | |
| 0 | + | 3-21 | 18.5 | 100 |
| 1.25 | + | 3-21 | 26.0 | 79 |
| 2.5 | + | 3-21 | 28.5 | 71 |
| 5 | + | 3-21 | 27.5 | 75 |
| CPA (5 μg/ml) | + | 3-21 | 64.5**[d] | 64 |
| 3 h treatment | | | | |
| 0 | - | 3-21 | 18.5 | 100 |
| 1.25 | - | 3-21 | 25.5 | 94 |
| 2.5 | - | 3-21 | 26.5 | 105 |
| 5 | - | 3-21 | 28.5 | 107 |
| MMC (0.125 μg/ml) | - | 3-21 | 148.0** | 80 |
| COL (0.2 μg/ml) | - | 3-21 | 58.5** | 54 |
| 24 h treatment | | | | |
| 0 | - | 24-0 | 21.0 | 100 |
| 1.25 | - | 24-0 | 29.5 | 86 |
| 2.5 | - | 24-0 | 29.0 | 87 |
| 5 | - | 24-0 | 27.5 | 105 |
| MMC (0.0625 μg/ml) | - | 24-0 | 155.0** | 89 |
| COL (0.2 μg/ml) | - | 24-0 | 117.0** | 40 |

[a]Time, Chemical treatment time-recovery time.
[b]2000 cells were examined per culture.
[c]RCC = (Cell counts of treated flask/Cell counts of untreated flask) × 100.
[d]Fisher's exact test; $^{**}p < 0.01$.
Abbreviation: RCC, Relative cell count; CPA, cyclophosphamide; MMC, mitomycin C; COL, colchicine.

tive controls (CPA, MMC and colchicine) included in the study could induce a statistically significant increase in the number of micronucleated cells when compared to negative control group. No cytotoxicity was observed whatever the concentration and the treatment schedule up to 5 μl/ml of WSF. Although there was a marginal increase in the number of micronucleated cells, the difference was not statistically significant (Table 3). Thus,

**Table 4.** *In vitro* comet assay for wood smoke flavors

| Conc. (μl/ml) | S9 mix | % Tail DNA[a] | Olive tail moment | Relative cell count (%)[b] |
|---|---|---|---|---|
| 3 h treatment | | | | |
| 0 | + | 7.86 ± 3.91 | 5.51 ± 3.23 | 100 |
| 1.25 | + | 9.68 ± 5.12 | 6.14 ± 3.21 | 83.2 |
| 2.5 | + | 9.91 ± 5.12* | 7.00 ± 4.85 | 86.5 |
| 5 | + | 13.04 ± 6.17** | 8.71 ± 4.82 | 75.8 |
| 3 h treatment | | | | |
| 0 | - | 7.41 ± 4.55 | 5.14 ± 5.15 | 100 |
| 1.25 | | 8.61 ± 5.81 | 6.00 ± 4.05 | 76.7 |
| 2.5 | - | 8.99 ± 5.17 | 6.75 ± 5.72 | 80.8 |
| 5 | - | 9.52 ± 7.21 | 6.88 ± 5.20 | 88.4 |
| 24 h treatment | | | | |
| 0 | - | 6.46 ± 3.85 | 4.41 ± 3.5 | 100 |
| 1.25 | - | 7.92 ± 5.84 | 5.40 ± 3.90 | 87.1 |
| 2.5 | - | 8.24 ± 5.13 | 6.14 ± 4.85 | 93.0 |
| 5 | - | 8.82 ± 5.93* | 7.32 ± 7.40 | 90.9 |
| Positive controls | | | | |
| CPA (10 μg/ml, 3 h) | + | 19.29 ± 12.54** | 11.51 ± 6.96 | 54.8 |
| $H_2O_2$ (200 μM, 30 min) | - | 88.59 ± 5.17** | 78.60 ± 15.37 | 71.1 |
| MMS (0.1 mM, 3 h) | - | 77.78 ± 6.15** | 48.54 ± 6.15 | 74.0 |

*$p < 0.05$, statistically significant vs. negative control according to Kruskal-Wallis test with post-test.
[a]100 cells were examined per culture.
[b]Viability measured by trypan blue exclusion; expressed as percentage of absorbance of control.

WSF was classified as negative in all treatment schedules without cytochalasin B up to 5 μl/ml.

***In vitro* comet assay.** To define *in vitro* comet assay doses, cytotoxicity is evaluated in terms of decrease in cell viability immediately after treatment, using the Trypan blue exclusion test. Giannotti *et al.* (2002) reported that, in some case, strand breaks are too short lived to allow detection after a 3 h treatment period due to preferential repair. Thus, comet assay was performed using a standard 3 h exposure time in both the absence and presence of S9 mix for short treatment. With WSF, cell growth was not significantly affected in the range of concentrations tested. The results of the alkaline comet assay with L5178Y $tk^{+/-}$cells were shown Table 4. Currently, there is no consensus on standard statistical methods for the analysis of comet data (Tice *et al.*, 2000). In this study, nonparametric *Kruskal-Wallis test* was used for statistical evaluations of % tail DNA. Slight but statistically significant increases in % tail DNA were sporadically found. However, this marginal increase was not concentration-dependent and none exceeded a maximal fold increase of 2.0. Thus, WSF was judged to be negative in all the treatment schedules. The positive controls treated with CPA and MMS caused significant ($p < 0.01$) increases in % tail moment as well as Olive tail moment.

## DISCUSSION

The pyrolysis of wood products yields a complex mixture of chemicals, which are often mutagenic and toxic. Several studies have characterized the mutagenicity of WSF in *Salmonella* mutagenicity assay. However, the available data is controversial. Two studies reported an increase in mutagenic activity for WSF (Asita *et al.*, 1991; Pool *et al.*, 1983) while other two studies found negative results (Braun *et al.*, 1987; Putnam *et al.*, 1999). These differences might be due to the type of wood used and the process of smoke production. Thus, it is of interest to determine whether wood smoke flavors prepared under domestic conditions contains detectable levels of cell mutagens. In this study, Ames test using *Salmonella typhimurium* strains showed that there was no significant increase in the number of revertant colonies compared to its negative control at any dose in all strains. The application of metabolic activation system (S9 mix) had little effect on the mutagenic potential of WSF. While a number of reports of WSF using the *Salmonella* assay were previously published, very little data are available in literature on the potential clastogenicity of WSF using other genotoxicity assays. This study showed that there was little clastogenicity seen in the *in vitro* chromosomal aberration assay. The analysis of standard genotoxicity assay data that has been conducted for safety evaluation of chemical compounds usually reveals a fairly high percentage of positive results associated with cytotoxicity in the *in vitro* chromosomal aberration assay (Galloway, 2000; Muller and Sofuni, 2000). No cytotoxicity was observed after the addition of liquid condensates of WSF up to 5000 μg/ml in CHL cells.

In addition to *in vitro* chromosomal aberration test, the *in vitro* micronucleus and alkaline (pH > 13) comet assays have been the most commonly practiced assays for the routine screening of potential genotoxicants (Kirsch-Volders *et al.*, 2003; Oliver *et al.*, 2006; Rojas *et al.*, 1999; Tice *et al.*, 2000) that utilize a variety of cell types that include L5178Y cell lines. The L5178Y cells were preferred for the present genotoxicity study because of the reasons that they have a short generation time and are easy to maintain. While the micronucleus test detects DNA lesions after their fixation into chromosome mutations, the alkaline version of the comet assay (Singh *et al.*, 1988) detects primary DNA single and double strand breaks and alkali-labile sites. In *in vitro* micronucleus test, the use of cytochalasin B is generally required to make sure analysed cells have completed a division (Fenech and Morley, 1985), especially in case of human lymphocytes that require a mitogenic stimulation to divide in culture. However, the need for cytochalasin B is still controversial for actively dividing cell lines. In addition, no obvious differences in the judgement for the compounds were seen whether cytochalasin B was used or not (Oliver *et al.*, 2006). The presence of cytochalasin B was shown to increase the levels of spontaneous micronucleated cells in the L5178Y cell line (Lorge *et al.*, 2006). European Centre for the Validation of Alternative Methods (ECVAM) also suggested that it does not need to be used with cell lines as long as cell proliferation is assured (Corvi *et al.*, 2008). WSF was classified as negative in all treatment schedules without cytochalasin B up to 5 μl/ml at which WSF produced low or no cytotoxicity although there was a marginal increase in the number of micronucleated cells.

Table 4 shows the % tail DNA and Olive tail moment obtained in the alkaline comet assay with WSF (1.25, 2.5 and 5 μl/ml). The International Workshop on Genotoxicity Test Procedures recommend the use of image analysis for comet single-cell gell electrophoresis assays, and give % tail DNA, tail length and tail moment as the recommended measurement parameters. Of these, there is much to recommend the use of % tail DNA, as this gives a clear indication of the appearance of the comets and is linearly related to the DNA break frequency over a wide range of levels of damage (Hartmann *et al.*, 2003), although tail moment measurement is the most commonly reported. These same recommendations were upheld at the International Workshop on Genotoxicity Test Procedures at San Francisco in 2005, with a recommendation that % tail DNA be the most suitable measurement parameter (Burlinson *et al.*, 2007). Thus, in this study, comet assay results were analyzed with % tail DNA. On the other hand, cell response to test article usually shows broad heterogeneity of values of % tail DNA at the single doses because comet assay examines each individual cell. Thus, the homogeneity of variances of data was tested with *Bartlett's test* ($p < 0.05$) and then, if the variances of data were not equal, nonparametric *Kruskal-Wallis test* was used for statistical evaluations. With this statistical analysis, WSF appeared to induce slight but statistically significant increases in % tail DNA. However, it is considered that there is no evidence of *biological relevance* because this marginal increase was not concentration-dependent and none exceeded a maximal fold increase of 2.0.

Our results lead to conclusion that WSF was judged to be negative based on four *in vitro* genotoxicity results. However, there are several publications showing the genotoxic potentials of WSF in genotoxicity assays using bacteria and lymphoblast cells. Thus, further experiments will be performed to confirm genotoxic effects of WSF with appropriate *in vivo* test system. In addition, the difference in genotoxicity data depending on WSF batch when they are manufactured raises the necessity of improved management system for the type of wood used and the process of smoke production.

## ACKNOWLEDGEMENTS

This research was supported by a grant (08152KFDA421) from Korea & Drug Administration in 2008, and the views presented in this article do not necessarily reflect those of the KFDA.
